# Essential oil-based hydrogels for oral candidiasis: preliminary formulation development, antifungal efficacy, and computational analysis via Monte Carlo simulation

**DOI:** 10.3389/fcimb.2026.1774370

**Published:** 2026-04-07

**Authors:** Adeola Tawakalitu Kola-Mustapha, Favour Temidayo John, Ronke Hadiyat Bello, Oluwakorede Joshua Adedeji, Yusuf Oluwagbenga Ghazali, Fahd Adebola Khalid-Salako, George Oche Ambrose

**Affiliations:** 1 College of Pharmacy, Alfaisal University, Riyadh, Saudi Arabia; 2 Faculty of Pharmaceutical Sciences, University of Ilorin, Ilorin, Nigeria; 3 Sabanci Universitesi, Istanbul, Türkiye; 4 University of Ilorin Teaching Hospital, Ilorin, Nigeria

**Keywords:** antifungal, *Cymbopogon flexuosus*, hydrogels, oral candidiasis, Syzygium aromaticum

## Abstract

**Introduction:**

This study aimed to evaluate the inhibitory activity of the essential oils (EOs) of *Syzygium aromaticum* (formerly *Eugenia caryophyllata*) (clove) and *Cymbopogon flexuosus* (lemongrass) on *Candida albicans* (individually, and in combination) and incorporate them into hydrogels for the management of oral candidiasis.

**Method:**

The antifungal activity of both essential oils singly and in combination was performed using macro and checkerboard dilution against a standard *C. albicans* (ATCC 10231) and 14 clinical isolates.

**Results and Discussion:**

Both EOs exhibited MIC of 100 μL/mL and MFC of 100 to 200 μL/mL depending on isolate; and a ΣFIC value of 0.75 indicating an additive outcome among 71% of the isolates. The EOs were formulated in hydrogels, the efficacies of which were tested using agar well diffusion method. The prepared hydrogels containing 3% w/w EOs in the ratio of 2:1 exhibited the best antifungal activity with desirably physicochemical and stability properties. Monte Carlo simulations (n = 10,000) further quantified the antifungal efficacy, revealing significantly enhanced inhibition arising from a synergistic effect of both EOs in combination. These findings highlight the promise of natural compounds as antifungal agents targeting critical fungal biosynthetic pathways and provide a basis for formulating EOs in hydrogels, potentially for the management of oral candidiasis.

## 1 Introduction

Several pharmaceutical dosage forms are available depending on the active ingredient’s site of action and administration method (Mahato and Narang, 2017). The protection of an active therapeutic molecule from early degradation, improvement of treatment efficacy, and reduction of side effects are the main objectives in the development of drug delivery systems. In an ideal scenario, controlled release devices can fulfil the requirements by decreasing dosage and frequency of administration while maintaining the drug concentration within a therapeutic window over an extended period. For many therapeutic compounds, oral drug delivery is the best method for administration, especially for chronic disorders. When compared to injection-based administration, the oral route is less complicated, increases patient compliance and comfort, and may even be less expensive (Liechty et al., 2010; Vermonden et al., 2012; Sharpe et al., 2014). However, if the medications can be administered directly to the lesion site, minimizing the systemic side effects, the effectiveness of the treatment can be increased (Magbool et al., 2020).

Successful delivery requires innovative drug delivery techniques to overcome obstacles such as enzymatic degradation and low bioavailability. One such technique is the protection offered by hydrogel networks. Crosslinked hydrophilic components make up the three-dimensional, polymeric networks known as hydrogels. Hydrogels can absorb significant amounts of water or biological fluids while remaining insoluble in specific environmental circumstances. Physical crosslinks, such as entanglements and crystallites, as well as chemical crosslinks, maintain the physical integrity of aqueous media (e.g., tie-points, junctions). Hydrogels have physical characteristics like those of biological tissues due to their high propensity for absorbing water, including soft consistency and low interfacial tension with aqueous fluids (Sharpe et al., 2014). These characteristics more closely resemble natural tissues than those of any other category of synthetic biomaterials, making them extremely biocompatible for use in biological applications. As such, the usage of hydrogels in drug delivery can be quite advantageous. Since they may be made anionic, cationic, or amphiphilic through the proper copolymerization processes with ionic components, hydrogels are crucial as carriers for oral delivery (Sharpe et al., 2014). Based on the widespread prevalence of oral microbial infections and importance of hydrogels, this project was designed to develop hydrogel for the treatment of oral candidiasis.

Oral mucosa is the term for the mucous membrane that covers the structures inside the boundaries of the oral cavity. Two layers make up this wet soft tissue membrane: the lamina propria, which is deeper, and the stratified squamous epithelium on the surface (Groeger and Meyle, 2019; Brizuela and Winters, 2024). The role of epithelial tissues is to shield the organism from physical, chemical, and microbiological threats. Being a diversified ecosystem, the oral cavity, which includes the teeth, gingiva, tongue, cheeks, and palates, can support a diversity of microorganisms that can occupy special ecological niches (Kilian et al., 2016). *Actinomyces, Arachnia, Bacteroides, Bifidobacterium, Eubacterium, Fusobacterium, Lactobacillus, Leptotrichia, Peptococcus, Peptostreptococcus, Propionibacterium, Selenomonas, Treponema*, and *Veillonella* are among the anaerobic bacteria found in the oral cavity (Sutter, 1984). Also present in the oral cavity are a variety of fungi, such as *Candida, Cladosporium, Aspergillus, Fusarium, Glomus, Alternaria, Penicillium*, and *Cryptococcus* (Cui et al., 2013). A condition caused by overgrowth of *Candida albicans* residing in the mouth is oral candidiasis, also known as oral thrush. That is, oral candidiasis is a mycosis (yeast/fungal infection) of Candida species on the mucous membranes of the mouth. Due to humans’ heightened vulnerability to fungal infections, the prevalence of fungal infections has steadily risen over time (Mota Fernandes et al., 2021; Mendonça et al., 2022). Oral candidiasis is the most prevalent type of candidiasis in humans and the most prevalent opportunistic oral infection. About two thirds of AIDS patients diagnosed with esophageal candidiasis concurrently present oral candidiasis (Yamada et al., 2011). Topical and systemic chemotherapeutic antifungal preparations are available to treat oral oropharyngeal candidiasis, and they have specific mechanisms of action that target sterols in cell membranes or enzymes involved in nucleic acid synthesis (Magbool et al., 2020). The preferred antifungal treatments for treating these infections, whether as topical applications or oral dosage forms, remain to be azole medications and their derivatives. Despite being highly acknowledged for their effectiveness, these medications can have negative side effects. Fluconazole belonging to the azoles, a medication with fungistatic properties, is becoming less effective against clinical isolates of *C. albicans* (Bondaryk et al., 2013). As a result, there is a need for therapeutic alternatives to treat yeast infections that are efficient and have minimal adverse effects (Suman et al., 2017).

When compared to synthetic antimicrobial chemicals, Natural products are often regarded as promising antimicrobial agents due to their diverse mechanisms of action and potential for reduced resistance development compared to conventional synthetic agents (Biernasiuk et al., 2022). Essential plant oils are fragrant oily liquids that may be extracted from different plant sections and are known to have a wide range of antibacterial properties. Different essential plant oils have been examined for *C. albicans in vitro* growth (Suman et al., 2017). The essential oil of *S. aromaticum* (SA) has been studied for its biological effects on a variety of microbes and parasites, including pathogenic bacteria, herpes simplex, and hepatitis C viruses. Clove essential oil has anti-inflammatory, cytotoxic, insect repellent, and anesthetic qualities in addition to its antibacterial, antioxidant, antifungal, and antiviral activity (Chaieb et al., 2007). In recent studies, clove essential oil has been seen to possess high antifungal activities against different yeast species (Suman et al., 2017). Furthermore, *C. flexuosus* (CF) is traditionally used for treating fever, stomach aches, headaches, diabetes, rheumatism, hypertension, wounds, bone fractures etc. The antimicrobial, antifungal, anticancer, anti-inflammatory, and analgesic characteristics of lemongrass essential oil have been found to be due to the high amount of the citral constituents, limonene, and geranyl acetate in them (Pathania et al., 2022).

Although these concentrations demonstrate effective antifungal activity, they represent relatively high exposure levels, further underscoring the importance of defining the therapeutic index through direct cytotoxicity assessment on oral epithelial cell models prior to clinical translation, as essential oil constituents such as eugenol and citral have been reported to exhibit concentration-dependent cytotoxic effects in mammalian cells (Chaieb et al., 2007; Bakkali et al., 2008).

In this study, the aim is to explore novel alternatives for the treatment of oral candidiasis. Accordingly, the main objectives are to design and develop a stable, efficacious, and safe local oromucosal drug delivery system of clove and lemongrass essential oils-loaded hydrogel. The hydrogels will be formulated to be used as an antifungal hydrogel against oral candidiasis.

In addition to conventional *in vitro* studies, computational modelling techniques have emerged as valuable tools in evaluating the antifungal potential of natural products, with statistical modelling providing evidence for synergistic or additive effects, where applicable. In this study, Monte Carlo simulation, a probabilistic method for analyzing variability in biological response was also deployed to ensure a robust estimation of essential oil efficacy under simulated conditions, thereby supporting the development of optimized formulations (Zriouel et al., 2023).

## 2 Materials and method

### 2.1 Media, chemicals and reagents

Mueller Hilton agar (MHA), Mueller Hilton broth (MHB), Sabouraud Dextrose Agar (SDA), Sabouraud Dextrose Broth (SDB), Brain Heart Infusion Broth (BHIB) all of Oxoid (Basingstoke, UK), Resazurin Dye (Canvax Biotech), Glycerol solution (Sigma – Aldrich^®^, Massachusetts, USA), Normal Saline (Juhel, Lagos, Nigeria), Distilled water (Central Research Laboratory, University of Ilorin, Kwara, Nigeria), (Oxoid, Basingstoke, UK), Carboxymethylcellulose, Poly Lactic Acid, Triethanolamine, Phosphate Buffer, Tween 80, *S. aromaticum* (SA) pure essential oil (Piping Rock, New York, USA) and *C. flexuosus* (CF) pure essential oil (Piping Rock, New York, USA).

### 2.2 Equipment

Water bath (Fischer Scientific Company, USA), Electronic analytical weighing balance (Ohaus), Incubator (Microfield: model SM9092, 240V and 2340W), Laminar air flow (Class II laminar flow Cellgard 220/240V), Autoclave (Portable 230V and 1850W XLife, India), Refrigerator (LG: Model No. HRF-688-FF/A), Vortex mixer (XH-C1, USA), Digital viscometer (Model NDJ 5S), pH meter (Hanna, UK), fixed and variable micropipettes (IndiaMART).

### 2.3 Ethical clearance

Ethical approval with the reference number ERC/MOH/2023/06/120 was granted by the Ethical Review Committee, Ministry of Health, Kwara State, Nigeria (**Supplementary Material File**). Prior to sample collection, permission, oral and informed consent were also sorted from the health care facility authority and the selected participants.

### 2.4 Study design

This study comprises of the following objectives as follows: (i) to determine the prevalence rate of oral candida among healthy individuals; (ii) to assess (*in vitro)* the antifungal susceptibility of oral candida using macro tube dilution method; (iii) to assess the resistance profile as well as phenotypes among candida isolates; (iv) to determine the minimum inhibitory and fungicidal concentrations of *S. aromaticum* (*E. caryophyllata)* and *C. flexuosus* essential oils using macro tube dilution methods; (v) to evaluate the synergistic interaction of both essential oils using checkerboard method; (vi) to formulate and characterize hydrogels prepared from *S. aromaticum* (*E.caryophyllata)* and *C. flexuosus* essential oils and determine their activity against isolated ora*l C. albicans*; (vii) To evaluate the ability of essential oil compounds to inhibit fungal cell wall synthesis, a critical target in antifungal therapy.

### 2.5 Study criteria

The inclusion criteria for this study include individuals who were willing to participate in the study, registered in the selected health center, with or without symptoms of oral candidiasis and must not have brushed their teeth 2 hours before sample collection. Patients who had been on any form of antifungal therapy within 3 months before the study commencement and those who were not willing or not available at the time of study were excluded.

### 2.6 Study population and sample collection

A total of 47 buccal swabs were collected from healthy human subjects based on the prevalence reported by (Stecksén-Blicks et al., 2015). These oral swabs were collected by medical personnel in accordance with protocols described in literature after obtaining the necessary consents and ethical clearance (Szymańska et al., 2016). With the aid of oral swab sticks, buccal swabs were obtained from the oral cavities of healthy human subjects. After collection, the handles of the oral swabs were cut-off using a pair of scissors whose blades were previously sterilized with 70% ethanol (in 5 mL of BHI Broth) in sterile McCartney bottles with aluminum screw caps. All collected oral swab samples were labelled and transported in cold chain to the Laboratory Unit of the Pharmaceutical Microbiology and Biotechnology Department at the University of Ilorin (Nigeria), for immediate analysis.

### 2.7 Isolation and identification of *Candida* isolates

This was performed according to the procedure described by Byadarahally and Rajappa (Byadarahally Raju and Rajappa, 2011). Collected swab samples in BHI Broth were transported to the laboratory in cold chain and immediately incubated at 37 °C for 18 hours in the laboratory. Post 18 hours aerobic incubation, sample bottles showing visible or no visible growth turbidity were sub-cultured on prepared SDA plates, incubated for another 18 hours at 37 °C. Plates that exhibited growth or no growth on SDA plates and in BHI broth after 48 hours of aerobic incubation were deemed as positive and negative samples respectively. Furthermore, colonial morphology on SDA plates were read and recorded appropriately. Additionally, Gram staining and Germ tube test were performed; large cream-colored, mucoid, pasty colonies, budded yeast-like cells occurring singly or in chains and filamentous extension of yeast cells after 3 hours incubation in human serum were identified as *C. albicans* using reference *C. albicans* (ATCC 10231) as control. All identified yeast isolates were stored in 25% glycerol supplemented with BHI Broth at 4 °C until needed (CLSI, 2018).

### 2.8 Antifungal susceptibility testing

Antifungal susceptibility profile of the *C. albicans* isolates were evaluated using modified Kirby-Bauer discs diffusion method (Rosco, 2011; CLSI, 2018). The antifungal discs (Mast Group, UK) of flucytosine (1 µg), fluconazole (25 µg) and Nystatin (50 µg) were used. Standardized 0.5 McFarland turbidity of freshly sub - cultured isolates of *C. albicans* with approximate yeast density of 1.2 x 10^8^ CFU/mL were inoculated aseptically onto Mueller-Hinton agar (MHA) containing 2% glucose and 0.5 µg/mL methylene blue. Aseptically, the discs were placed at equidistance on the inoculated plates. The MHA plates were allowed to pre-diffuse for 30 minutes and incubated at 37 °C for 24 hours. After 24 h incubation, the inhibition zones were measured in millimeter (Cheesbrough, 2006), and interpreted based on the recommendations of Rosco (2011).

### 2.9 Antifungal screening of essential oil *S. aromaticum* and *C. flexuosus*

This was performed using the macro broth dilution method according to the protocol described in literature (Ayinde et al., 2023). A total of 15 C*. albicans* isolates comprising 14 clinical isolates and a reference strain *C. albicans* ATCC 10231. A total 10 tubes each were used for all isolates, six (6) tubes contained varying concentrations of 100 – 600 µL/mL in 1 mL MH broth while four (4) were control tubes. These control tubes include the viability (Tube 7: organism + 1 mL MH Broth), positive (Tube 8: 50 µg/mL nystatin + 1 mL of MH Broth), broth sterility (Tube 9: 1 mL MH Broth only), essential oils control (Tube 10: 100 µL of either of the essential oils + 1 mL of MH Broth) control tubes. Then, standardized inocula (100 µL) of 0.5 McFarland turbidity was introduced into Tube 1 to 7 using a micropipette —bringing the final volume to 50 - 300 µL/mL, mixed by gentle upward and downward movement and incubated for 24 hours at 37 °C. After incubation, all tubes were observed macroscopically for visible turbidity. Concentrations without visible turbidity were deemed as concentration of essential oils of *S. aromaticum and C. flexuosus* exhibits antifungal activity.

#### 2.9.1 Determination of minimum inhibitory concentrations of *S. aromaticum* and *C. flexuosus* essential oils

Using a 96-well plate (Corning, NY, USA), concentration range of 1.56 – 400 µL/mL was prepared by serial dilution in 100 µL MHB across well 1–9 for each *C. albicans* isolates running through row A – H. For each isolate, control wells (10 -12) were included, namely, organism viability, broth sterility, and essential oil sterility control. Standardized inocula (100 µL) of 0.5 McFarland turbidity of each *C. albicans* isolates was introduced into well A – H across well 1–10 using a micropipette, to yield a final concentration of 0.78 – 200 µL/mL. Prepared plates were incubated for 24 hours at 37 °C. After 24 hours aerobic incubation, 100 μL of 10% resazurin dye was aseptically added to each well and plates were incubated at 37 °C for an hour. Resazurin dye is a microbial growth indicator that binds to metabolically active yeast cells and as such reduce blue resazurin to pink product called resorufin. The lowest concentration where there is no visible color change from blue to pink was regarded as the MIC.

#### 2.9.2 Determination of minimal fungicidal concentrations of *S. aromaticum* and *C. flexuosus* essential oils

Content in the MIC well and concentrations higher than the MIC was sub-cultured on prepared MHA plates and incubated at 37 °C for another 24 hours. Post incubation, the concentration without growth after incubation period was taken as the Minimal Fungicidal Concentration (MFC).

#### 2.9.3 Determination of synergism in combinations of *S. aromaticum* and *C. flexuosus*

This was performed using the checkerboard synergism method as described by Altun and colleagues (Altun and Yapici, 2022). A hundred (100 µL) of two times the MIC of *S. aromaticum* and *C. flexuosus* was used as the starting concentrations and serially diluted across both x and y-axis respectively using 100 µL of sterile MH Broth to give varying concentrations in a descending order hence creating a checkerboard sequence. Standardized 0.5 McFarland turbidity (100 µL) of the test Candida isolates was placed into all wells except the column 10–12 which were the control wells. Column 9 for organism viability, column 10 for broth sterility, column 11, 12 for essential oil sterility wells and all prepared plates were incubated for 24 hours at 37 °C. After 24 hours, 100 μL of 10% resazurin dye was aseptically added to all wells and incubation at 37 °C for an hour and the lowest concentration alone and in combinations where there were no color changes were used to determine the fractional inhibitory concentration index (FICI).

The ΣFICs were calculated following Equations 1–3:

(1) ΣFIC= FIC A (EC) + FIC B (CF)

(2) FIC A=MIC (combination)MIC (EC)

(3) FIC B=MIC (combination)MIC (CF)

The combination of the two EOs was determined according to the protocol of Limsrivanichakorn et al. (2020), as: synergistic when the FICI value is ≤ 0.5; additive when FICI ≥ 0.5 to ≤ 1; indifferent when FICI ≥ 1 to ≤ 2 and antagonistic when FICI ≥ 2.

### 2.10 Formulation of hydrogel

Pure essential oils of SA and CF were incorporated as active ingredients. Carboxy methyl cellulose (CMC) and poly lactic acid (PLA) were used to prepare the hydrogel, which contained 1% (v/v) and 2% (v/v) SA and CF pure essential oils respectively. While PLA is not a typical hydrogel base material, it was incorporated to provide controlled release of the essential oils, otherwise prone to rapid diffusion or volatilization from the hydrophilic gel structure, while also providing structural support and controlling swelling within the aqueous oral mucosal environment.

#### 2.10.1 Preparation of hydrogel base

Carboxy methyl cellulose (CMC) hydrogel was prepared by dispersing 3% carboxymethylcellulose powder in distilled water and letting it hydrate for 24 h to produce H1. PLA was also prepared by dispersing 3% PLA in distilled water to produce H2. Using the same method, several combinations of CMC and PLA polymers were made to select the best ratio that gave a suitable hydrogel. The PLA dispersion was transferred into the CMC formed gel after 24 h, at different mixing ratios (1:3, 1:1, 3:1 CMC: PLA) at the same temperature under continuous stirring. Within this mixture, the hydrophobic PLA, existing in dispersed fine microparticulate form, is immobilized within the highly viscous CMC gel network by steric effects, further stabilized by the subsequent addition of tween-80 which reduces tension at the hydrophilic-hydrophobic interfaces. These CMC-PLA mixtures were coded H3, H4, and H5 (**Table 1**). The samples were stored at room temperature for further analysis.

**Table 1:** Quantitative composition of hydrogel base.

Ingredients	H1	H2	H3	H4	H5
Carboxymethyl cellulose (g)	3	–	3	1	1
Poly lactic acid (g)	–	3	1	1	3
Water (g) to	100	100	100	100	100

#### 2.10.2 Preparation of essential-oils-loaded hydrogels

Using the method described in 2.10.1, the optimized hydrogel base (plain hydrogel) H3 was prepared. Tween 80, an emulsifier was added to stabilize the essential oils in dispersion within the hydrogel. Formulations H6, H7, H8 (**Table 2**) contain 2% (v/v) SA, 1% (v/v) CF, and 3% SA + CF, respectively were formed. The resulting complex hydrogels were adjusted to pH 6.86 with phosphate buffer near the pH of oral cavity (5.5 to 7). The samples were stored at room temperature for further analysis.

**Table 2:** Quantitative composition of different loaded hydrogel formulations.

Ingredients	H3	H6	H7	H8
Carboxymethyl cellulose (g)	3.0	3.0	3.0	3.0
Poly lactic acid (g)	1.0	1.0	1.0	1.0
Clove essential oil (SA) (g)	–	2.0	–	2.0
Lemongrass essential oil (CF) (g)	–	–	1.0	1.0
Tween 80 (g)	–	10	10	10
Water (g) to	100	100	100	100

### 2.11 Characterization of hydrogel

#### 2.11.1 Sensory properties and physical observations of hydrogel formulations

Unloaded hydrogel formulations were visually inspected for clarity, color, odor, consistency, and presence of particles. To investigate the consistency of the formulations, a small quantity of hydrogel was pressed between the thumb and the index fingers, and the consistency of the hydrogel was noticed. To check for grittiness, all the formulations were evaluated microscopically for the presence of any appreciable particulate matter which was seen under light microscope (Kaur, 2013). *SA* and *CF* hydrogel formulations were also visually inspected for clarity, color, odor, consistency, and presence of particles.

#### 2.11.2 Determination of pH

The pH influences the taste and stability of hydrogels. The pH of prepared essential oils loaded hydrogels were measured using a digital pH meter (HANN PH 209, Romania) at room temperature 25 °C ± 5 °C. For this purpose, 0.5 g of hydrogel was dispersed in 50 mL of distilled water to make a 1% solution. The pH of each formulation was done in triplicate (Kostrzębska, 2023).

#### 2.11.3 Measurement of spreadability

*In-vitro* spreadability test was performed on the prepared essential oils-loaded hydrogel formulations to simulate their spread on the oral mucosal surface. The spreadability of the hydrogels was determined by placing a predetermined weight of samples (0.5 g) in between two glass slides of equal weight and area. The initial spreading diameter before placing the weight was noted. Thereafter, 100 g-weight was placed over the upper slide. The final diameter (in cm) was noted over a period of 60 secs. The percentage spreadability of the formulations was determined using Equation 4 (Uloma et al., 2021):

(4) $$\text{\% }Spreadability=\frac{Final\;Diameter-Initial\;Diameter}{Initial\;Diameter}\times 100\text{ \%}$$

#### 2.11.4 Measurement of extrudability

The area of the extruded hydrogel was measured after 10 g of the hydrogel were put into collapsible tubes and squeezed by applying 1 kg of load weight to the tube’s folded end, measuring the emulgel ribbon’s length, The test was performed in triplicate. The extrudability is then calculated by applying Equation 5: (Baibhav et al., 2012):

(5) $$Extrudability=\frac{Weight\;applied\;(g)}{Surface\;area\;of\;extruded\;ribbon\;(cm^{2})}$$

#### 2.11.5 Determination of viscosity

The viscosity of the hydrogel was measured at 25 ± 2 °C and 50 rpm using the DV3T Brookfield viscometer (Ametek Brookfield, USA) spindle 4. Every measurement recorded was the average of three determinations, presented as mean ± SD.

#### 2.11.6 Determination of homogeneity

The hydrogels were all tested for homogeneity by visual inspection after having been left to congeal and set in containers. They were examined for their appearance and presence of aggregates.

### 2.12 Evaluation of antifungal activity of prepared essential oils-loaded hydrogel

The agar well diffusion method as described by Kola-Mustapha et al. was used to screen the anti-candida activity of prepared *S. aromaticum* and *C. flexuosus* loaded hydrogel formulations (Kola-Mustapha et al., 2023). Standardized inocula of 0.5 McFarland turbidity was inoculated on MHA by swabbing. Then, sterile cork borer with 6 mm diameter was used to punch wells in prepared MHA plates, sealed with a few drops of molten MHA and 100 µL of formulated hydrogels (plain hydrogel (H3), SA alone (H6), CF alone (H7), SA and CF hydrogel (H8) previously solubilized in 2% Tween -80. All plates were incubated at 37°C for 24 h, after which the diameters (mm) of inhibition were measured using a meter rule and regarded as zones of inhibition (ZOI).

### 2.13 Empirical Monte Carlo and bootstrap analysis of MIC/MFC data

To quantitatively evaluate the variability and robustness of the fungicidal response observed in the minimum inhibitory concentration (MIC) and minimum fungicidal concentration (MFC) assays, a non-parametric empirical Monte Carlo framework was implemented using the observed dataset from 14 clinical isolates of *Candida albicans*. All isolates exhibited a uniform MIC of 100 μL/mL for both *Syzygium aromaticum* and *Cymbopogon flexuosus* essential oils. However, MFC values varied between 100 and 200 μL/mL across isolates.

The probability of fungicidal activity at the MIC concentration was defined as the proportion of isolates for which MIC equaled MFC. To estimate uncertainty around this probability without imposing parametric distributional assumptions, bootstrap resampling was performed with 10,000 iterations using the boot package in R (version 4.3.0). Percentile-based 95% confidence intervals were computed from the re-sampled distributions.

In addition, empirical Monte Carlo resampling was conducted by drawing 10,000 simulated MFC values with replacement from the observed dataset. This approach preserves the discrete structure of the experimental outcomes and allows probabilistic estimation of future isolate behavior based solely on empirical frequencies. All statistical analyses and visualizations were performed in R using the packages dplyr, ggplot2, and boot (Patil et al., 2015; Mushi et al., 2017; Vila et al., 2020; Kola-Mustapha et al., 2023).

#### 2.13.1 Statistical analysis

Descriptive statistics, including mean, standard deviation, and 95% confidence intervals (CIs), were calculated for each treatment group. Group differences in simulated antifungal activity were analyzed using one-way analysis of variance (ANOVA), followed by Tukey’s Honestly Significant Difference (HSD) test for pairwise comparisons. Homogeneity of variance was assessed using Levene’s Test, and if violated, non-parametric Kruskal-Wallis tests with Dunn’s *post-hoc* comparisons were conducted. All statistical tests were conducted at a significance level of p < 0.05. Effect size was determined using Cohen’s d to assess the magnitude of differences between treatment groups, with interpretations based on standard thresholds (small: 0.2, medium: 0.5, large: 0.8). Bayesian analysis was also performed using Bayes Factor (BF) estimation to provide additional evidence for the treatment effect.

A sensitivity analysis was conducted by varying the assumed mean inhibition values of clove EO from 70 to 80% in 1% increments, while keeping the standard deviation constant, to assess the impact on simulated mean inhibition outcomes. Additionally, the probability that the EO combination outperformed the individual oils was estimated by calculating the proportion of simulations where the combination’s inhibition values were greater than those of clove EO and lemongrass EO, respectively.

#### 2.13.2 Unimodality testing

To investigate the distribution characteristics of the simulated data, Hartigan’s Dip Test for unimodality was applied to each treatment group using the *‘dip.test ()’* function in R. This test assessed whether the distributions were unimodal or exhibited evidence of multimodality.

#### 2.13.3 Software and tools

All simulations and statistical analyses were conducted in R (version 4.3.0), using packages including ggplot2, dplyr, boot, car, FSA, effectsize, BayesFactor, and diptest.

## 3 Results

### 3.1 Distribution of oral *Candida* in healthy individuals

**Figure 1:**
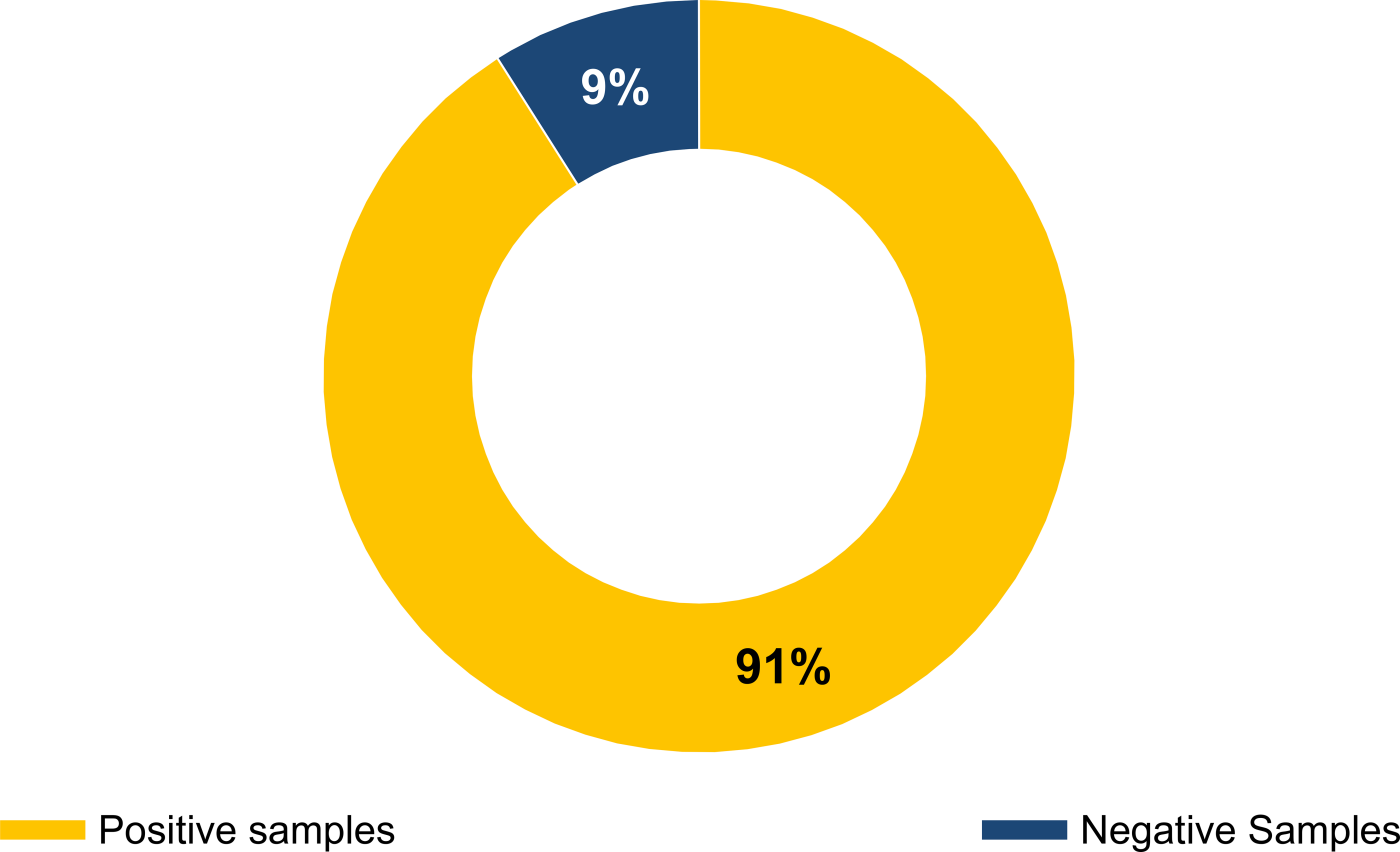
Distribution of oral candida among healthy individuals.

**Figure 1** shows the distribution of oral candida among 47 oral swabs in healthy individuals. Of the 47 collected swabs, 43 (91%) were positive, while 4 (9%) were negative cultures. Isolates from the 43 positive individuals were identified as *C. albicans* using the criteria shown in **Table 3**.

**Table 3:** Criteria for identification of Candida isolates from oral swabs.

S/no	Identification criteria	Characteristics
1	Colonial Morphology	White to off-white in color, circular, smooth, and mucoid-like colonies
2	Gram Reaction	Gram positive, budded medium or large oval cells
3	Germ Tube test	Filamentous or tube- like extension of yeast cells

### 3.2 Antifungal susceptibility testing of *Candida albicans* isolates

**Figure 2:**
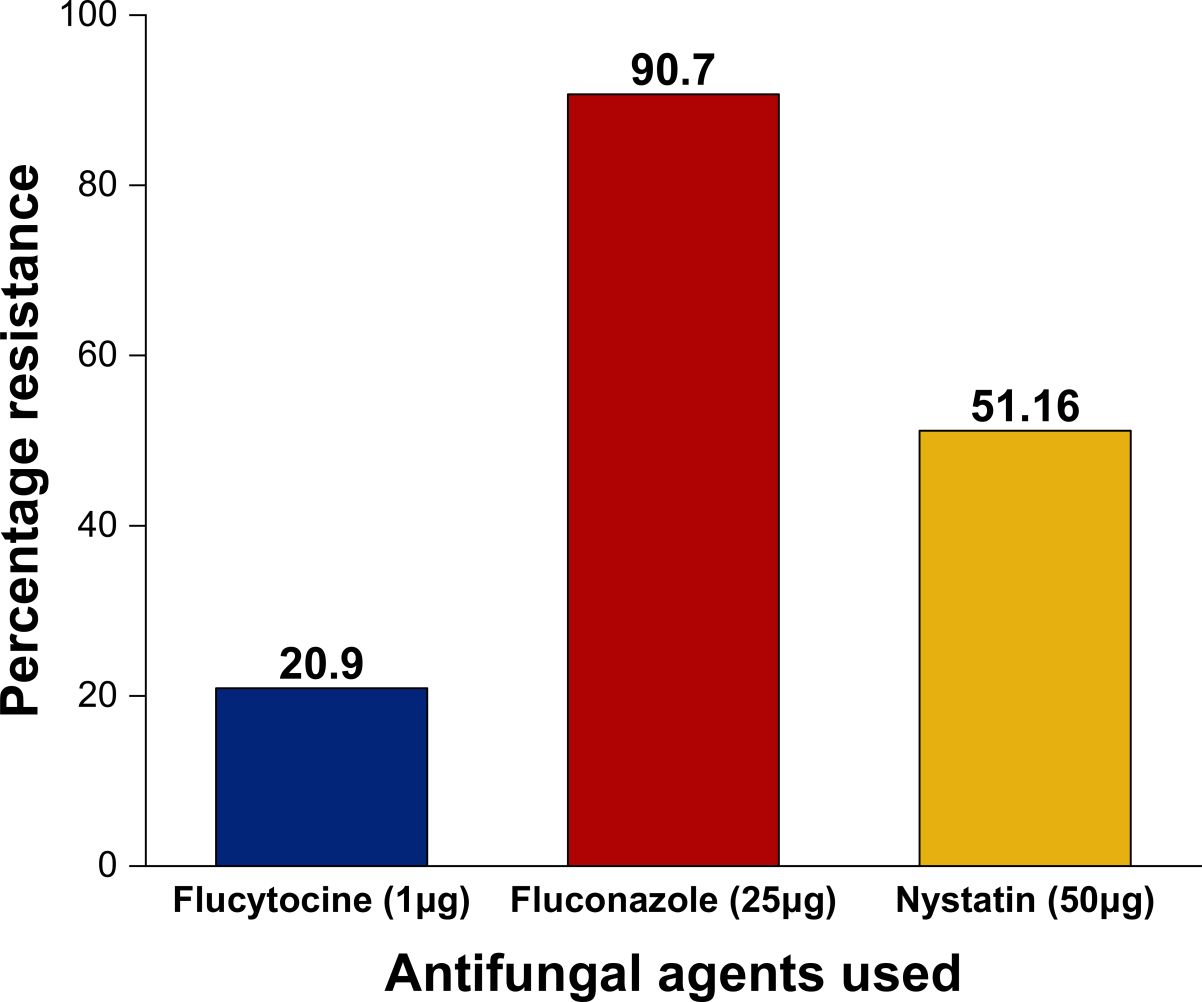
Antifungal resistance profile of *C. albicans* against selected antifungal agents.

**Figure 2** shows the antifungal resistance profile for 43 identified *C. albicans* with isolates exhibiting the highest resistance against fluconazole (39, 90.7%) and flucytosine being the least at (9, 20.9%). Isolates B08, B017 and B030 were sensitive while only B011 exhibited resistance to all the selected anti-fungal agents, generally exhibiting four (4) resistant phenotypes with FLU-NY being the most dominant at 52.5% as indicated in **Table 4**.

**Table 4:** Antifungal resistance pattern of *C. albicans* isolates.

S/no	Resistance pattern	F (%)
1	FLU	11 (27.50)
2	FY-FLU	7 (17.50)
3	FLU-NY	21 (52.50)
4	FY-FLU-NY	1 (2.50)
	TOTAL	40 (100)

Keys:

FY, Flucytosine (1 µg)

FLU, Fluconazole (25 µg)

NY, Nystatin (50 µg)

### 3.3 Minimum inhibitory concentration and minimum fungicidal concentration of *S. aromaticum* and *C. flexuosus*

**Table 5** shows the MIC and MFC of *S. aromaticum* and *C. flexuosus* against 14 selected *C. albicans* isolates exhibiting resistance to 2 or more of the different antifungal agents using Nystatin 50 µg/mL and *C. albicans* ATCC 10231 as control.

**Table 5:** MIC and MFC of *S. aromaticum* and *C. flexuosus* against *C. albicans* isolates.

Isolates codes	MIC (µl/mL)	MFC (µl/mL)	
	SA/CF	SA	CF
B001	100/100	200	100
B004	100/100	200	100
*B005	100/100	100	100
B006	100/100	200	200
*B011	100/100	200	100
B016	100/100	200	200
B019	100/100	200	200
B023	100/100	200	200
*B026	100/100	100	*100
B027	100/100	200	200
B028	100/100	200	200
B036	100/100	200	200
B039	100/100	200	100
*B043	100/100	100	100

*Isolates exhibiting MIC and MFC at same concentration for either SA or CF. C. albicans ATCC 10231: Positive control at 50 µg/mL of Nystatin. CF density – 0.893 g/mL; SA density – 1.039 g/mL.

### 3.4 Synergistic action of *S. aromaticum* and *C. flexuosus* oils in combination

#### 3.4.1 Checkerboard bioassay

**Table 6** shows the results of the checkerboard bioassay carried out to confirm the synergistic interactions of both essential oils in combination against selected *Candida albicans* isolates.

**Table 6:** Results from the checkerboard bioassay against selected *Candida albicans* isolates.

Isolates codes	MIC Alone		FIC	ΣFIC	Interpretation
	SA (µL/mL)	CF (µL/mL)			
B001	100	100	50/25	0.75	Additive
B004	100	100	100/50	1.5	Indifference
B005	100	100	50/25	0.75	Additive
B006	100	100	100/50	1.5	Indifference
B011	100	100	50/25	0.75	Additive
B016	100	100	50/25	0.75	Additive
B019	100	100	100/50	1.5	Indifference
B023	100	100	50/25	0.75	Additive
B026	100	100	50/25	0.75	Additive
B027	100	100	50/25	0.75	Additive
B028	100	100	100/50	1.5	Indifference
B036	100	100	50/25	0.75	Additive
B039	100	100	50/25	0.75	Additive
B043	100	100	50/25	0.75	Additive

*C. albicans* ATCC 10231: ΣFICs = FICA + FICB = 0.75 (Additive).

### 3.5 Characterization of the formulated hydrogels

#### 3.5.1 Results obtained from the physical characterization of the formulated hydrogels

This characterization was done by examining the organoleptic properties of the hydrogels, with results presented in **Table 7**.

**Table 7:** The organoleptic properties of the formulated hydrogels.

Properties	H3	H6	H7	H8
Appearance	Smooth	Smooth	Smooth	Smooth
Color	White	Cream	Cream	Cream
Odor	Odorless	Characteristic Clove Smell	Intense, lemon-like	Lemon and clove smell
Texture	Smooth	Smooth	Smooth	Smooth
Homogeneity	Good	Good	Good	Good
Skin irritation	Non-irritant	Non-irritant	Non-irritant	Non-irritant
Ease of application	Easy application	Easy application	Easy application	Easy application
Ease of Removal	Easy removal with water	Easy removal with water	Easy removal with water	Easy removal with water

#### 3.5.2 pH, spreadability, extrudability and viscosity of the hydrogels

The results of pH, spreadability and extrudability of the formulated hydrogels are presented in **Table 8**.

**Table 8:** The pH, Spreadability extrudability and viscosity of the formulated hydrogels.

Hydrogels	pH (mean ± SEM)	Spreadability (cm)	Extrudability (g/cm^2^)	Viscosity (cP)
H3	6.86 ± 0.03	6.2 ± 0.1	380 ± 15	2250 ± 50
H6	6.85 ± 0.03	5.7 ± 0.2	400 ± 18	2850 ± 70
H7	6.85 ± 0.04	5.9 ± 0.2	395 ± 17	2700 ± 65
H8	6.86 ± 0.02	5.4 ± 0.1	410 ± 20	3050 ± 80

#### 3.5.3 Stability testing of the hydrogels

The formulated gels were evaluated for their stability after one week, one, two and three months. The results of physical evaluation over this period revealed that there was no significant change in the color, odor, texture, appearance, homogeneity, and skin irritation tendencies of the formulated hydrogels. The physical properties of the hydrogels at the end of three months (post formulation) are presented in **Table 9**.

**Table 9:** Physical evaluation after three (3) months at 25 ± 2°C.

Properties	H3	H6	H7	H8
Appearance	Smooth	Smooth	Smooth	Smooth
Color	White	Cream	Cream	Cream
Odor	Odorless	Characteristic Clove smell	Intense, lemon-like smell	Clove + Lemon like smell
Texture	Smooth	Smooth	Smooth	Smooth
Homogeneity	Good	Good	Good	Good
Skin irritation	Non-irritant	Non-irritant	Non-irritant	Non-irritant

#### 3.5.4 Anti-candida activity of the hydrogels

**Figure 3:**
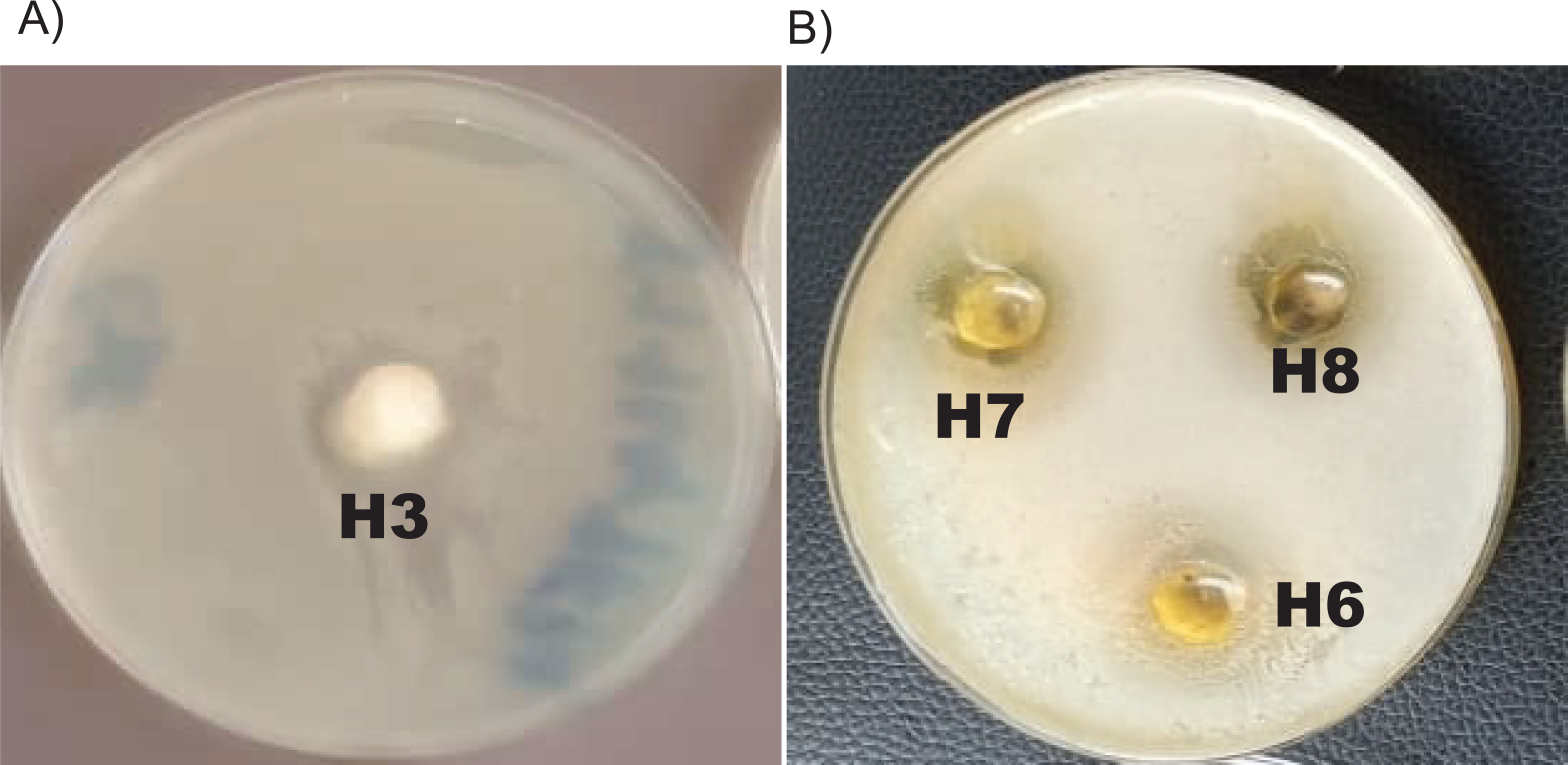
Antifungal activity of hydrogel formulations as presented by zones of inhibition: **(a)** H3, plain hydrogel; **(b)** H6, *C. flexuosus* hydrogel; H7, *S. aromaticum* hydrogel; H8, combination of *S. aromaticum* and *C. flexuosus* hydrogel.

The formulated hydrogels showed different degrees of inhibitions against *C. albicans* strain as presented in **Table 10** and **Figure 3**.

**Table 10:** Degree of inhibition of formulated hydrogels against *C. albicans.*.

		Zones of Inhibitions (mm)		
Organisms	H3	H6	H7	H8
B011	0.00	7.00	11.00	12.00
B039	0.00	8.00	10.00	12.00
B06	0.00	7.00	10.00	10.00
*Candida* (10231)	0.00	8.00	10.00	12.00
B04	0.00	8.00	11.00	12.00

Key: Diameter of cork borer = 6 mm, Positive control = Nystatin 50 µg/mL = 16 mm.

### 3.6 Growth inhibition of essential oils against fungal pathogens

All 14 clinical isolates demonstrated growth inhibition at 100 μL/mL, confirming uniform inhibitory activity of both essential oils at this concentration. The distribution of MFC values showed that three isolates exhibited fungicidal activity at 100 μL/mL, whereas eleven isolates required 200 μL/mL for fungicidal effect. This corresponds to an observed probability of fungicidal activity at the MIC concentration of 0.214.

Bootstrap resampling yielded a point estimate of 0.214 for the probability of MIC equaling MFC. The percentile-based 95% confidence interval ranged from 0.000 to 0.429, reflecting sampling variability inherent to the dataset.

**Figure 4:**
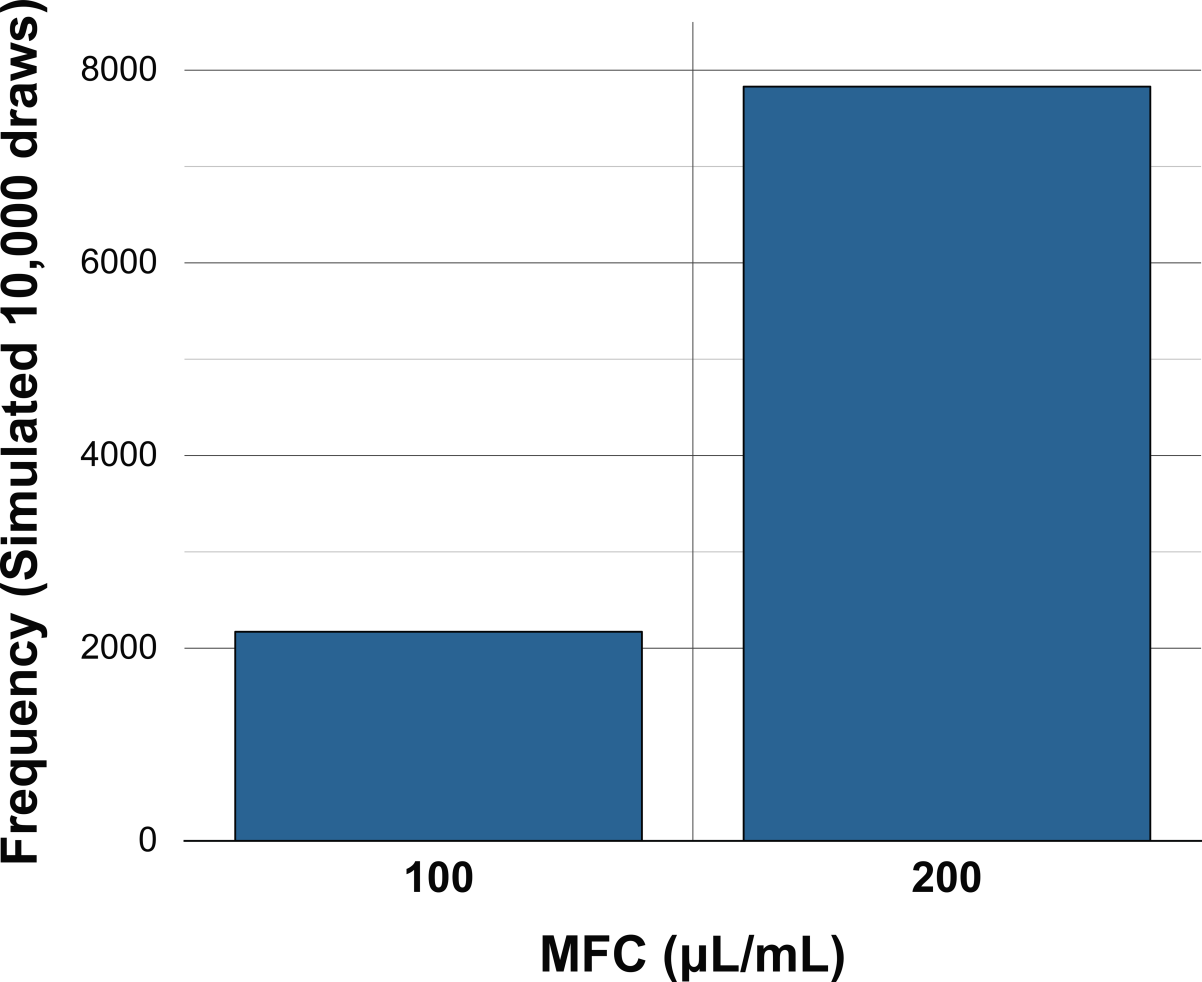
Empirical Monte Carlo simulation of minimal fungicidal concentration (MFC) distribution for *Candida albicans* clinical isolates (n = 10,000 resamples).

Empirical Monte Carlo resampling of 10,000 simulated isolates produced an estimated probability of 0.217 for MFC = 100 μL/mL and 0.783 for MFC = 200 μL/mL. The simulated distribution closely mirrored the observed frequencies, indicating stability of the empirical probability estimates. The resulting distribution is presented in **Figure 4**.

## 4 Discussion

Due to humans’ heightened vulnerability to fungal infections, the prevalence of fungal infections including oral candidiasis has steadily risen over time (Mota Fernandes et al., 2021; Mendonça et al., 2022). Oral candidiasis (OC) often called “oral thrush” is an infection of the oral cavity including the tongue, oropharynges and oral mucosal sites which is marked by candida or non-candida growth and invasion of superficial tissues (Vila et al., 2020). *C. albicans* remains the etiologic agent of asymptomatic OC in healthy human carriers and among immunocompromised persons especially in human immunodeficiency virus cases in sub-Sahara Africa (Mushi et al., 2017). Our findings showed *C. albicans* as the predominance fungi isolated from the buccal cavities of healthy individual suggesting it as the primary etiology in oral candidiasis. This result is relatively comparative to the findings in India and Poland who documented *C. albicans* as the presiding isolates among healthy carriers (Naidu and Reginald, 2016; Szymańska et al., 2016). This may be attributable to poor oral hygiene, prolonged use of antimicrobial mouth washes, smoking, high intake of refined sugar and poor nutritional status among subjects (Patil et al., 2015). The effective treatment of oral candidiasis depends on certain important factors such as the nature of localized infection, presence of underlying infections, immune status and the isolated candida species (Garcia-Cuesta et al., 2014). To understand the antifungal resistance trend for effective treatment and management oral candidiasis in the study population, antifungal susceptibility testing was carried out. Resistance to fluconazole was 90.7%, followed by nystatin (51.16%), flucytosine (20%) exhibiting high combined antifungal resistance phenotype between azoles and polyenes (FLU-NY) at 52.5%. This finding is in tandem with a study carried out in Mexico which reported 94.7% (Monroy-Pérez et al., 2016), while it differs from findings in a South African study which reported a lower rate of 56.7% fluconazole resistance (Dangor et al., 2014). This high fluconazole resistance within the study area is likely stems from the overuse of fluconazole in the empirical treatment of *Candida* species related infections. The increasing resistance to the azoles, especially the fluconazole, a first member of the azole group, poses serious health threat due to its common used for superficial and systemic candidiasis (Garcia-Cuesta et al., 2014). This pattern of resistance can also be linked to a homozygous ERG3 genes mutation which encodes a sterol C5, 6-desaturase in *C. albicans* strain (McTaggart et al., 2020).

In this study, flucytosine was the most sensitive (80%) antifungal agent. Other similar reports from Kenya and Slovakia among oral *C. albicans* infections corroborate with our findings (Bii et al., 2002; Černáková et al., 2022). Flucytosine (5-fluorocytosine, 5-FC) is a fluorinated derivative of the pyrimidine, cytosine, typically used in the treatment of candida–oriented infections (Patil et al., 2015). It is converted to 5-flurouracil and competes with uracil to inhibit protein synthesis and incorporate into fungal DNA and RNA, thus leading cell lysis (Delma et al., 2021). Recently, resistance to flucytosine was reported *in vitro* among 3–10% of *C. albicans* isolates with up to about 30% of resistance manifestation occurring during flucytosine monotherapy (Sigera and Denning, 2023). This can be said to justify the 20% resistance observed in this study. Despite resistance patterns and observed side effects, flucytosine is still recommended in treatment of different types of candidiasis especially when used in combination with amphotericin B or azoles to increase synergistic activity (van der Voort, 2008). To improve the quality of human life and overcome resistance, therapeutic effects of the essential oils of *S. aromaticum* and *C. flexuosus* were evaluated individually and in combination with optimal presentation as a suitable pharmaceutical product. It has previously been reported that *E.caryophyllata* has a lot of health advantages such as antimicrobial activity (El-Darier et al., 2018; Behbahani et al., 2019), antioxidant activity (Marmouzi et al., 2019), insecticidal activity (Elzayyat et al., 2018; Neupane et al., 2020; Reuss et al., 2020), antiviral activity (Lane et al., 2019), antinociceptive activity (Beltrán-Villalobos et al., 2017), and analgesic activity (Khalilzadeh et al., 2016). *C. flexuosus* has also been identified to have definite antimicrobial, antifungal, anticancer, analgesic, antioxidant, and cholesterol reduction properties (Mirghani et al., 2012; Rajeswara Rao, 2013). Prior to this time, the essential oils have been individually investigated for their clinical uses and have been used traditionally (in their crude forms) for various purposes (Chaieb et al., 2007; Ganjewala and Gupta, 2013; Suman et al., 2017; Pathania et al., 2022).

In demonstrating their anti-candida effects, the essential oils were individually subjected to antifungal screening, and the process was repeated for the combination of the essential oils as well. The physicochemical properties of the essential oils were also examined. The minimum inhibitory concentration (MIC) of both *C. flexuosus* and *S. aromaticum* was found to be 100 µL/mL, corresponding to 89.3 mg/mL and 104 mg/mL respectively. Although these concentrations demonstrate effective antifungal activity, they represent relatively high exposure levels, further underscoring the importance of defining the therapeutic index through direct cytotoxicity assessment on oral epithelial cell models prior to clinical translation. The results obtained agree with earlier findings in literature (Chaieb et al., 2007; Suman et al., 2017; Pathania et al., 2022), where it has been reported that *S. aromaticum* and *C. flexuosus* possess *in vitro* antifungal activity against the causative microorganism incriminated in the pathogenesis of oral thrush. Both *S. aromaticum* and *C. flexuosus* essential oils were observed to be active against multiple strains of *C. albicans* isolated from oral swabs.

The checkerboard bioassay was used to investigate the synergistic, additive, or antagonistic effect of the essential oils in combination. The results obtained were interpreted using fractional inhibitory concentration index (FIC). The FIC of each drug was calculated using the MIC of the drug in combination divided by the MIC of that drug alone. Significantly higher inhibition was observed with the combination than either SA or CF in individual antimicrobial studies, similar to literature findings (Chasanah et al., 2023; Shalihah et al., 2023). For all the wells of the microtiter plates that corresponded to an MIC, the sum of the FICs (ΣFIC) was calculated for each isolate. The 2:1 MIC combinations FIC values of SA to CF indicated a calculated ΣFICs of 0.75 for 71.43% of the isolates hence indicating additivity according to Meletiadis et al. (2010). Accordingly, the 2:1 combination ratio was used in the formulation of the hydrogel in this research.

The hydrogel was produced from carboxymethylcellulose and poly lactic acid after preparing them in varied ratios and subjecting them to further analysis to select the ratio that best suits the intended purpose. The formulated hydrogel exhibited acceptable organoleptic properties and preliminary compatibility under observational assessment, easy to apply and remove, which are desirable attributes of a topical dosage form. Other evaluations such as mucoadhesive strength and drug release kinetics are also important for the further development of this hydrogel system. Measuring mucoadhesive strength will help determine how well the formulation can stay attached to mucosal tissue for a sufficient time. This is important for effective drug delivery. In addition, studying the drug release kinetics will help show how the drug is released from the hydrogel over time. These evaluations will be important in future studies to better understand the behavior of the formulation and support its potential clinical use.

The optimized hydrogel base selected was H3 based on preliminary studies. For the physicochemical properties, the pH of the hydrogel formulated was measured to be 6.86 ± 0.02, which is acceptable considering the pH of the oral cavity. The acceptable pH range to avoid irritation to the oral cavity is 5.5-7 (Magbool et al., 2020), and the hydrogels pH fall within this range. This indicates that the hydrogels may not cause pH-induced irritation to the oral cavity. The incorporation of *C. flexuosus* and *S. aromaticum* essential oils into hydrogel formulations (H6, H7, H8) influenced their physicochemical properties in **Table 8** compared to the plain hydrogel base (H3). The addition of essential oils and Tween 80 resulted in a decrease in spreadability across formulations. H3 exhibited the highest spreadability (6.2 ± 0.1 cm), while H8 showed the lowest (5.4 ± 0.1 cm). This reduction is likely due to increased viscosity and the formation of a more complex gel network upon oil incorporation. Similar findings were reported where the inclusion of lemongrass oil in hydrogels led to decreased spreadability due to enhanced gel rigidity (Swetha et al., 2018). An increase in extrudability was observed in oil-loaded formulations, with H8 demonstrating the highest value (410 ± 20 g/cm²). This enhancement may be attributed to the plasticizing effect of Tween 80 and the lubricating properties of the essential oils, facilitating easier extrusion. These observations align with the work of Kumari and Bajpai, who noted improved extrudability in emulgels containing essential oils (Swetha et al., 2018). The viscosity of the hydrogels increased with the addition of essential oils and Tween 80, with H8 showing the highest viscosity (3050 ± 80 cP). This increase can be attributed to the formation of a more entangled polymer network and the presence of emulsified oil droplets, which enhance the internal resistance to flow. Aldawsari and his co-workers also reported increased viscosity in hydrogels upon incorporation of lemongrass oil-loaded nano sponges (Swetha et al., 2018). The incorporation of clove and lemongrass essential oils, stabilized by Tween 80, modified the hydrogel’s physicochemical properties, enhancing viscosity and extrudability while reducing spreadability. These modifications are consistent with previous studies and suggest potential for improved therapeutic efficacy in oral applications.

The stability evaluation of the formulated hydrogels (H3–H8) over a three-month period at 25 ± 2 °C demonstrated consistent physical characteristics, including appearance, color, odor, texture, homogeneity, and skin compatibility. These findings align with previous studies on essential oil-based hydrogels, indicating that such formulations can maintain their integrity and user acceptability over extended storage periods. The stability over three months supports the physicochemical robustness of the formulation and its suitability for further biological evaluation. The results obtained in this research show the potentiated antifungal effects of both essential oils when used in combination of 2:1 rather than individually, which establishes their potential in the clinical management of oral thrush caused by *C. albicans*.

The incorporation of computational modelling into this study offers new insights into the antifungal potential of essential oil combinations, reinforcing the emerging role of *in silico* methods in natural product research. The application of Monte Carlo simulations allowed for the robust evaluation of variability in antifungal efficacy, providing predictive strength to the observed synergistic effects of *Syzygium aromaticum* and *Cymbopogon flexuosus* essential oils. This probabilistic approach complements traditional bioassays by quantifying the likelihood of therapeutic success across a range of biological variability, a critical factor in clinical translation. The observed additive trends align with previous findings which highlight the enhanced antimicrobial activity of essential oil combinations due to multifaceted mechanisms of action, including membrane disruption, enzyme inhibition, and interference with fungal biofilms (Soulaimani, 2025). Furthermore, the use of statistical modelling, including effect size estimation and Bayesian analysis, underscores the clinical relevance of these combinations, offering a quantitative basis for formulating EO-based interventions. This integrated approach supports the growing consensus that natural product synergy can be systematically studied and optimized, rather than empirically observed alone (Caesar and Cech, 2019). Importantly, such findings advocate for a paradigm shift in antifungal therapy development, where computational predictions guide the design of more effective, targeted, and patient-specific treatments. In the context of rising antifungal resistance, particularly among *Candida albicans* isolates, the ability to predict and confirm synergistic efficacy through both *in vitro* and *in silico* methods represents a significant advancement in the search for novel, natural-based therapeutics.

In general, the findings presented in this study demonstrate that both *Syzygium aromaticum* (formerly *Eugenia caryophyllata*) and *Cymbopogon flexuosus* essential oils exhibit consistent inhibitory activity against clinical isolates of Candida albicans, with a uniform MIC of 100 μL/mL across all tested isolates, corroborating previous reports of strong antifungal activity of clove and lemongrass oils against Candida species (Chaieb et al., 2007; Negrelle and Gomes, 2007; Freire et al., 2017). The antifungal efficacy of clove oil is largely attributed to eugenol, which disrupts fungal cell membranes, alters permeability, and interferes with ergosterol biosynthesis, while lemongrass oil, rich in citral, exerts membrane-destabilizing and oxidative stress–inducing effects (Chaieb et al., 2007; Negrelle and Gomes, 2007). Despite consistent growth inhibition, fungicidal activity exhibited variability, with only 21.4% of isolates demonstrating MFC values equal to the MIC and 78.6% requiring 200 μL/mL for fungicidal effect. This divergence between inhibitory and fungicidal thresholds is consistent with established antifungal pharmacodynamics, where growth suppression does not necessarily translate to fungal cell death (Meletiadis et al., 2010). Variability in fungicidal response may reflect strain-specific differences in membrane composition, stress-response pathways, efflux pump expression, and ergosterol biosynthesis alterations, all of which have been implicated in antifungal tolerance and resistance among *C. albicans* isolates (Bondaryk et al., 2013; Delma et al., 2021). The probabilistic framework applied in this study, using non-parametric bootstrap resampling and empirical Monte Carlo simulation, demonstrated that the likelihood of achieving fungicidal activity at 100 μL/mL is approximately 0.21, while the probability of requiring 200 μL/mL is substantially higher, reflecting biologically plausible inter-isolate heterogeneity. Such variability aligns with global reports of differing antifungal susceptibility patterns across clinical settings (Dangor et al., 2014; Monroy-Pérez et al., 2016). From a translational perspective, these findings underscore the importance of sustained local drug concentrations to achieve fungicidal outcomes, supporting the development of mucoadhesive hydrogel systems capable of prolonged drug residence and controlled release within the oral cavity (Liechty et al., 2010; Sharpe et al., 2014). Given the rising prevalence of azole resistance among Candida species and the need for alternative therapeutic strategies (Monroy-Pérez et al., 2016; Delma et al., 2021), essential oil–based formulations may offer a promising adjunctive approach. Although the sample size in the present study is modest, the integration of empirical resampling methods strengthens the robustness of the probability estimates and provides a statistically grounded interpretation of antifungal potency, reinforcing the potential of essential oil–loaded hydrogels for localized management of oral candidiasis.

Importantly, although *in silico* analyses are useful in the prediction of potential interactions at the molecular level, these predictions do not always fully translate to measurable synergistic inhibition of bacterial proliferation *in vitro*. Docking assumes an ideal binding and does not capture factors such as membrane permeability, efflux pumps, metabolic stability, or whole cells environment as with checkerboard assay. According to Pan et al. (2023), *in silico* for drug combination effects are significant models for prioritizing drug candidates but have inherent limitations when comparing with phenotypic responses, such as checkerboard assays. More so, Guan et al. (2022) demonstrated that *in silico* analysis such as molecular docking can suggest probable mechanisms of interaction that must be confirmed by biological assays such as checkerboard assay to determine the influenced of biological characteristics. Therefore, a synergistic prediction by docking can be consistent with additive outcomes in checkerboard assays when complex biological influences are considered. Having established the antifungal potential of *S. aromaticum* and *Cymbopogon flexuosus* essential oils in hydrogel formulations, future research directions will incorporate *in vitro* cytotoxicity evaluation on oral mucosal cell lines. While literature evidence supports the antifungal efficacy of the essential oils, their safety profiles are influenced by concentration, exposure duration, and formulation matrix. The present investigation focused primarily on antifungal activity, physicochemical characterization, and computational modelling. Future studies will incorporate standardized cytotoxicity assays (e.g., MTT or resazurin-based viability assays) using human oral keratinocytes and fibroblasts to determine the therapeutic index of the optimized hydrogel formulation. Such evaluation is essential before progression toward preclinical or clinical application.

## 5 Conclusions

This study successfully formulated and evaluated novel oral hydrogels incorporating clove (*S. aromaticum*) and lemongrass (*C. flexuosus*) essential oils, stabilized with Tween 80, for potential antifungal application against Candida species. The antifungal activity of *C. flexuosus* and *S. aromaticum* against *C. albicans*, (the causative agent in oral candidiasis), is increased when used in combination. An additive effect was experienced when the essential oils were combined in the ratio of 2:1. The optimized formulations (H6, H7, H8) demonstrated favorable physicochemical characteristics, including suitable spreadability, extrudability, and viscosity, for oral topical delivery.

The stability assessments over a three-month period confirmed that all formulations retained their physical integrity, organoleptic properties, and non-irritant nature, indicating their potential for safe and effective use in the oral cavity. The incorporation of essential oils not only provided natural antimicrobial properties but also contributed to the overall structural and sensory performance of the gels. These findings suggest that essential oil-loaded hydrogels offer a promising alternative for the localized management of oral candidiasis, contributing to an emergent focus on standardized natural pharmaceuticals-based formulations as an approach to addressing systemic side effects, antimicrobial resistance, and patient adherence, among other drawbacks of conventional antimicrobials.

## Data Availability

The original contributions presented in the study are included in the article/**Supplementary Material**. Further inquiries can be directed to the corresponding author.
