# Supplementary material for: Essential oil-based hydrogels for oral candidiasis: preliminary formulation development, antifungal efficacy, and computational analysis via Monte Carlo simulation
Source: Front Cell Infect Microbiol. 2026 Apr 7;16:1774370. doi: 10.3389/fcimb.2026.1774370 (PMC13096092; doi:10.3389/fcimb.2026.1774370)
Supplement: Supplementary file 1 [file Table1.docx]

Supplementary Material

# Supplementary Data

**
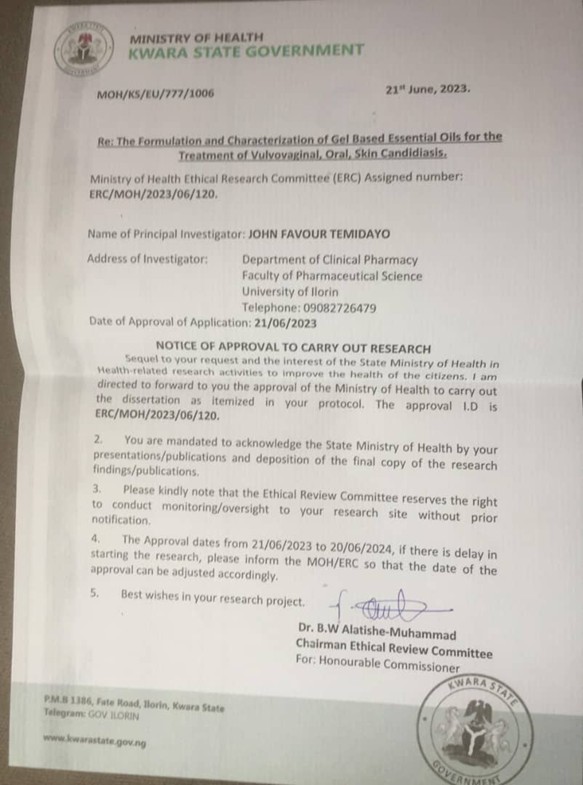
**

**Supplementary Figure 1.** Ethical Approval Document.
